# Effect of acupuncture treatment on vascular cognitive impairment without dementia: study protocol for a randomized controlled trial

**DOI:** 10.1186/1745-6215-15-442

**Published:** 2014-11-13

**Authors:** Bo-Feng Yang, Xiang-Hong Zeng, Yan Liu, Qing-Nan Fu, Tian He, Fang Li, Guang-Xia Shi, Bao-Zhen Liu, San-Feng Sun, Jun Wang, Lei Xiao, Yan-Mei Deng, Cun-Zhi Liu

**Affiliations:** Acupuncture and Moxibustion Department, Beijing Hospital of Traditional Chinese Medicine affiliated to Capital Medical University, 23 Meishuguanhou Street, Dongcheng District, Beijing, 100010 China; Tianjin University of Traditional Chinese Medicine, 312 Anshan West Road, Nankai District, Tianjin, 300193 China; Beijing Huairou District Hospital of Traditional Chinese Medicine, 1 Houheng Street jia, Huairou District, Beijing, 101400 China; Dongzhimen Hospital Affiliated to Beijing University of Traditional Chinese Medicine, 5 Haiyuncang, Dongcheng District, Beijing, 100010 China; Beijing Fengtai Hospital of Integrative Medicine, 60 Changxindian Dangsanposanli jia, Fengtai District, Beijing, 100072 China

## Abstract

**Background:**

Vascular cognitive impairment, no dementia (VCIND) is a condition at risk for future dementia and should be the target of preventive strategies. Preliminary evidence suggests that acupuncture may be a clinically effective intervention for people with early-stage vascular cognitive impairment. We will do a multicenter, 6-month, drug-controlled, nonblinded, randomized, parallel-group trial to determine whether acupuncture is effective for improving cognitive function and quality of life for patients with VCIND.

**Methods/Design:**

A total of 216 eligible patients will be recruited and randomly assigned acupuncture for two sessions/week (n = 108) or citicoline 300 mg/day (n = 108) in a multicenter, 6-month trial. The primary endpoint is cognition (Alzheimer's Disease Assessment Scale, Cognitive Subscale (ADAS-cog)). Secondary endpoints include assessments of activities of daily living and behavioral symptoms (Clock Drawing Test (CDT), Activities of Daily Living (ADL) and Instrumental Activities of Daily Living scale (IADL)).

**Discussion:**

This will be the first large-scale trial specifically evaluating acupuncture therapy in VCIND. If the study confirms the effectiveness and safety of acupuncture treatment, it will be important to examine how the acupuncture approach could most effectively be integrated into the provision of routine healthcare.

**Trial registration:**

This study is registered as an International Standard Randomised Controlled Trial on 17 January 2014, number ISRCTN 82980206

**Electronic supplementary material:**

The online version of this article (doi:10.1186/1745-6215-15-442) contains supplementary material, which is available to authorized users.

## Background

Cerebrovascular disease is the second most common cause of cognitive disorders [1]. Vascular cognitive impairment (VCI) reflects cognitive disorders that are associated with vascular disease [2]. Some stroke-related factors such as multiple small or large strokes and ischaemic white-matter lesions have been associated with poststroke VCI. Six months after stroke, as many as 44 to 74% of patients present some degree of cognitive disturbance [3–5]. Stroke patients with cognitive impairment but no dementia have an increase in the 5-year risk of developing dementia of any type [6]. VCI with no dementia (VCIND) is a potentially treatable and preventable cause of dementia in later life, and familiarity with this condition will help the practitioner provide better care to patients [1]. Acupuncture is widely used for patients with cognitive disorders. It has been confirmed that acupuncture has positive effect on cognition and quality of life in patients who had a stroke [7]. However, there is no data on the effectiveness and safety of acupuncture used for VCIND patients. The aim of this study is to assess the effectiveness and safety of acupuncture in the treatment of VCIND patients.

## Methods/Design

### Design

**Figure 1:**
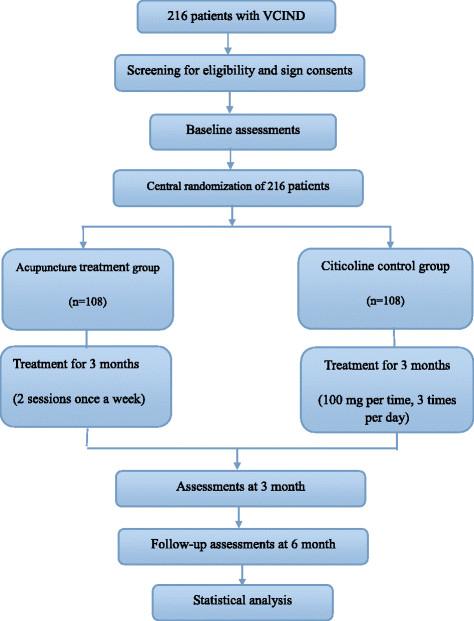
**Flow chart.**

The design is a three-center randomized trial (Figure 1). Two hundred and sixteen patients with VCIND will be recruited for inclusion in the study upon fulfillment of the selection criteria. The patients will be randomly allocated into the acupuncture treatment group (two sessions/week for 3 months) or the citicoline (Qilu Pharmaceutical Co., Ltd., Shandong, China) control group (100 mg/time, three times/day for 3 months). There will be a 3-month follow-up period. General ethical approval has been obtained from the ethics committee of the Beijing Hospital of Traditional Chinese Medicine affiliated to Capital Medical University on 22 March, 2013 (Ref: 201317) (Additional file 1).

### Participants

Patients will be recruited in acupuncture clinics in the Beijing Hospital of Traditional Chinese Medicine Affiliated to Capital Medical University, the Beijing Chinese Medicine Hospital in Huairou District, the Beijing Fengtai Hospital of Integrative Medicine and the Dongzhimen Hospital Affiliated to Beijing University of Chinese Medicine.

### Inclusion criteria

Patients who meet all of the following conditions will be considered for enrollment. The inclusion criteria are as follows: subjects (males and females) age 55 to 85 years old; fluency in language sufficient to reliably complete all study assessments; a Hachinski score ≥7; evidence of vascular lesions based on neuroradiology; a Mini-Mental State Examination (MMSE) score > dementia threshold corrected according to the years of education (score >17, score >20 and score >24 for 0, ≤6, and >6 years of education, respectively); a Montreal Cognitive Assessment (MoCA) score <25 for ≤12 years of education or score <26 for >12 years of education; and signing of an informed consent form.

### Exclusion criteria

The exclusion criteria are as follows: history of mental diseases (for example, schizophrenia, serious anxiety and depression); Alzheimer's disease (AD), Parkinson's disease (PD), frontotemporal dementia (FTD) or Huntington's disease (HD); epilepsy and/or ever having antiepileptic drugs; or the presence of serious heart disease, kidney disease or liver disease.

### Interventions

After giving informed consent and completing a baseline evaluation, each participant will be randomized to the acupuncture treatment group or to the citicoline control group. The acupuncture treatment strategies are developed by consensus with experienced acupuncture practitioners and a neurologist. The acupuncture group will receive acupuncture at the Zusanli (ST36), Xuehai (SP10), Danzhong (RN17), Zhongwan (RN12), Qihai (RN6), Baihui (DU20), Fengfu (DU16), Xinshu (BL15), Yixi (BL45), Tongli (HT5) and Zhaohai (KI6) acupuncture points. Only sterile, stainless, single-use needles (Hwato, Suzhou, China) without guide tubes will be used. The gauge is 0.35 × 40 mm or 0.35 × 25 mm and the depth of insertion is 4 to 50 mm, depending on the location of the needle. Needles are correctly inserted and manually stimulated until the ‘De Qi’ sensation is elicited. ST36, SP10, RN17, RN12, RN6, and DU16 will be inserted 10 to 15 mm deep with a 0.35 mm × 40 mm acupuncture needle. DU20, BL15, BL45, HT5 and KI6will will be inserted 5 to 10 mm deep with a 0.35 mm × 25 mm acupuncture needle. The needles are retained for 20 minutes in every session. Other forms of acupuncture treatment (for example, laser acupuncture, electro-acupuncture or moxibustion) will not be permitted.

The citicoline control group will receive oral citicoline 100 mg/time, three times/day. A systematic review of the literature has revealed that citicoline is safe and effective in improving poststroke cognitive decline [8].

Treatment will be conducted over a period of 3 months, at a frequency of two sessions per week of acupuncture treatment group and three times per day for the citicoline control group.

### Education of acupuncturists

The participating acupuncturists will be members who have been qualified for at least 3 years, hold a Chinese medicine practitioner license from the Ministry of Health of the People’s Republic of China and take an educational course to ensure their strict adherence to the study protocol and familiarity with the administering study.

### Randomization

After signed informed consent and baseline measurements have been obtained, randomization will be performed according to a random list of numbers generated with SAS software (SAS Institute, Inc., Cary, NC, USA). The allocation list will be handled by an independent investigator who has no contact with the study participants and is not involved in the supervision of staff responsible for the collection of data. The allocation of the randomization sequence will be concealed centrally by telephone.

### Blinding

The interviewers who measured the outcomes will be blinded to the randomization status. It is not possible to blind participants due to the nature of the intervention. Blinding also will be maintained for data management, outcome assessment and data analysis.

### Primary outcome

A general cognitive performance battery will be evaluated using the Alzheimer's Disease Assessment Scale-Cognitive (ADAS-Cog). ADAS-cog is a quantitative instrument designed to assess the severity of cognitive impairment over time in Alzheimer’s disease patients, but it still is widely used for the cognitive assessment in mild to moderate vascular dementia [9]. The Chinese version of the ADAS-cog subscale has been determined to be reliable and valid among older Chinese in Hong Kong [10]. The adapted scale consists of 13 individual tests, including word recall, naming objects and fingers, commands, constructional and ideational praxis, orientation, word recognition, spoken language and comprehension, word finding and recall of test instructions. The ADAS-Cog requires approximately 30 to 45 minutes to administer, depending on the degree of cognitive impairment [11].

The ADAS-Cog will be assessed at baseline (before treatment initiation), the end of treatment and 3 months after the end of treatment.

### Secondary outcome measures

Cognition of the executive function and praxis will be evaluated using the Clock Drawing Test (CDT). Executive impairments have been found to be the most common functioning impairments following stroke [12]. The CDT assesses cognition, focusing on executive function and praxis in contrast to the more language-based Mini-Mental State Examination (MMSE) [13].

The longitudinal functional outcome will be evaluated using the Ability of Daily Living (ADL) and Instrumental Activities of Daily Living (IADL). The functional ability of patients with vascular cognitive impairment is gradually lost [14]. Measures of functional disability typically contain items that reflect limitations in performing the ADL or IADL. Combining IADL and ADL items together in the same scale would provide enhanced range and sensitivity of measurement [15].

The CDT, ADL and IADL will be assessed at baseline (before treatment initiation), the end of treatment and 3 months after the end of treatment.

### Other outcomes

The perceived credibility of treatment will be evaluated by the Treatment Credibility Scale (TCS) [16]. It is a five-item questionnaire ranging from 1 (not at all) to 5 (very confident). Items are averaged to provide a single treatment credibility score, with high scores reflecting high treatment credibility.

Adverse events (AEs) will be actively assessed by the trial physicians using a list at each session of acupuncture, including discomfort or bruising at the sites of needle insertion, nausea, or feeling faint after each acupuncture treatment. Adverse drug reactions like nausea and vomiting, dizziness, dry mouth, and itching will also be recorded.

The TCS and AEs will be assessed at the end of treatment.

Concomitant drugs will be recorded during the study, including those taken for the control of vascular risk factors (hypertension, diabetes, atherosclerosis, atrial fibrillation, APOE, homocysteine, etcetera), antithrombotic treatments for cardioembolism, and the use of antiplatelet agents for noncardioembolic stroke.

### Sample size

According to the previous study, an improvement of ADAS-Cog scores in the treatment group and the placebo group were 1.8 (standard deviation: 5.94) and 0.3 (standard deviation: 6.32), respectively [17]. The difference in scores between the two groups was 1.5. Accordingly, we assume that a difference within 1.5 indicates acupuncture is not inferior to citicoline treatment. Using a non-inferiority design, and assuming a power of 80%, an alpha value of 5%, and a population standard deviation of 5.89, the required sample size is 94 patients in each group. Allowing for 15% attrition, we should recruit 216 patients, with 108 in each group.

### Data analysis

Descriptive statistics will be used to describe demographic and baseline characteristics of study participants. A generalized estimation equation (GEE) will be used to test the between-group differences. The accepted level of significance for all analyses will be *P* <0.05. Data analysis will be conducted by statisticians who are independent of the research team. Analysis will be conducted using SPSS software (SPSS 12.0 KO for Windows ©).

## Discussion

Acupuncture is a part of Traditional Chinese Medicine and has been used for thousands of years to treat clinical disorders, including stroke-related deficits. However, the possible effects of acupuncture on cognitive function have received little attention or the evidence base is poor. Acupuncture may be effective in treating some neurogenic cognitive or communicative disorders such as autism [18]. The problems associated with cognition issues after stroke continue to be challenged. This study is expected to provide evidence, with high external validity, for the effectiveness and safety of acupuncture treatment on VCIND patients. To our knowledge, this is the first large-scale, multicenter, randomized, controlled trial specifically evaluating acupuncture therapy in VCIND.

The MMSE is a brief and widely used test for screening for cognitive impairment; however, it has been criticized as a poor screening test for VCIND due to insensitivity to visuospatial and executive function impairments commonly involved in patients with cerebrovascular disease [19, 20]. The Montreal Cognitive Assessment (MoCA) evaluates multiple domains of cognition, including visuo-executive, naming, attention, language, abstraction, delayed recall and orientation, but cannot completely replace the MMSE [21]. In our study, global cognitive function will be assessed using the MMSE and the MoCA in the initial screening session.

The trial has some limitations. First, in certain circumstances, it may be difficult to determine whether the cognitive decline is based on vascular factors, Alzheimer’s disease, or both (mixed forms) because AD and VCI can co-occur and interact in a manner [22, 23]. Second, acupuncturists and participants cannot be blinded to the group allocation, although all outcome measures will be administered and collected by an independent researcher to minimize the risk of detection bias. We will carefully discuss residual sources of bias and their potential impact on clinical outcomes when we analyze the study findings.

These results from this trial will provide preliminary evidence about the usefulness and acceptability of acupuncture for patients with VCIND, which will serve as a basis for further research.

## Trial status

This trial is currently recruiting participants.

## Electronic supplementary material

Additional file 1: **Ethical approval.** (DOC 4 MB)

## Abbreviations

ADAS-cog: Alzheimer's Disease Assessment Scale, Cognitive Subscale
ADL: Activities of Daily Living
AEs: adverse events
CDT: Clock Drawing Test
IADL: Instrumental Activities of Daily Living Scale
MMSE: Mini-Mental State Examination
MoCA: Montreal Cognitive Assessment
TCS: Treatment Credibility Scale
VCIND: vascular cognitive impairment, no dementia.
