# Supplementary material for: Effect of acupuncture treatment on vascular cognitive impairment without dementia: study protocol for a randomized controlled trial
Source: Trials. 2014 Nov 13;15:442. doi: 10.1186/1745-6215-15-442 (PMC4242470; doi:10.1186/1745-6215-15-442)

**Ethical issues review application form**

| **Sponsor** | Beijing Municipal Science & Technology Commission |
| --- | --- |
| **ID number** | 201317 |

| **Number of should present** | **Number of actually present** | **Number of absent** |
| --- | --- | --- |
| 9 | 6 | 3 |

| **Opinion** | **poll** |
| --- | --- |
| Agree | 6 |
| Agree after correction | 0 |
| Disagree | 0 |
| Terminate or suspend this trial | 0 |

Decisions of the ethics review boards were made by a majority of the members present and voting in accordance with the Declaration of Helsinki. The study protocol of “Effect of acupuncture treatment on vascular cognitive impairment without dementia: study protocol for a randomized controlled trial” was approved.

The Research Ethical Committee of Beijing Traditional Chinese Medicine Hospital Affiliated to Capital Medical University


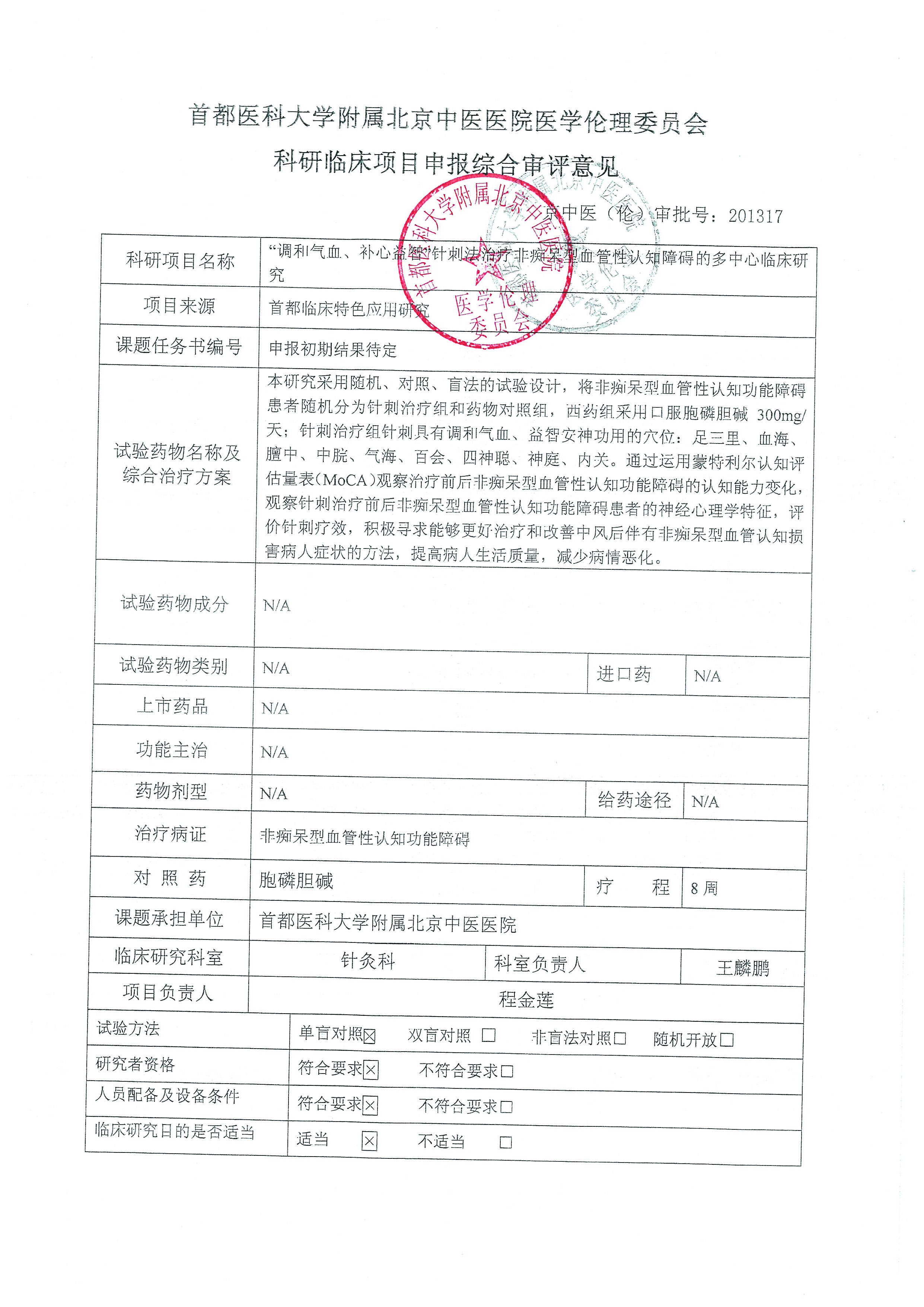


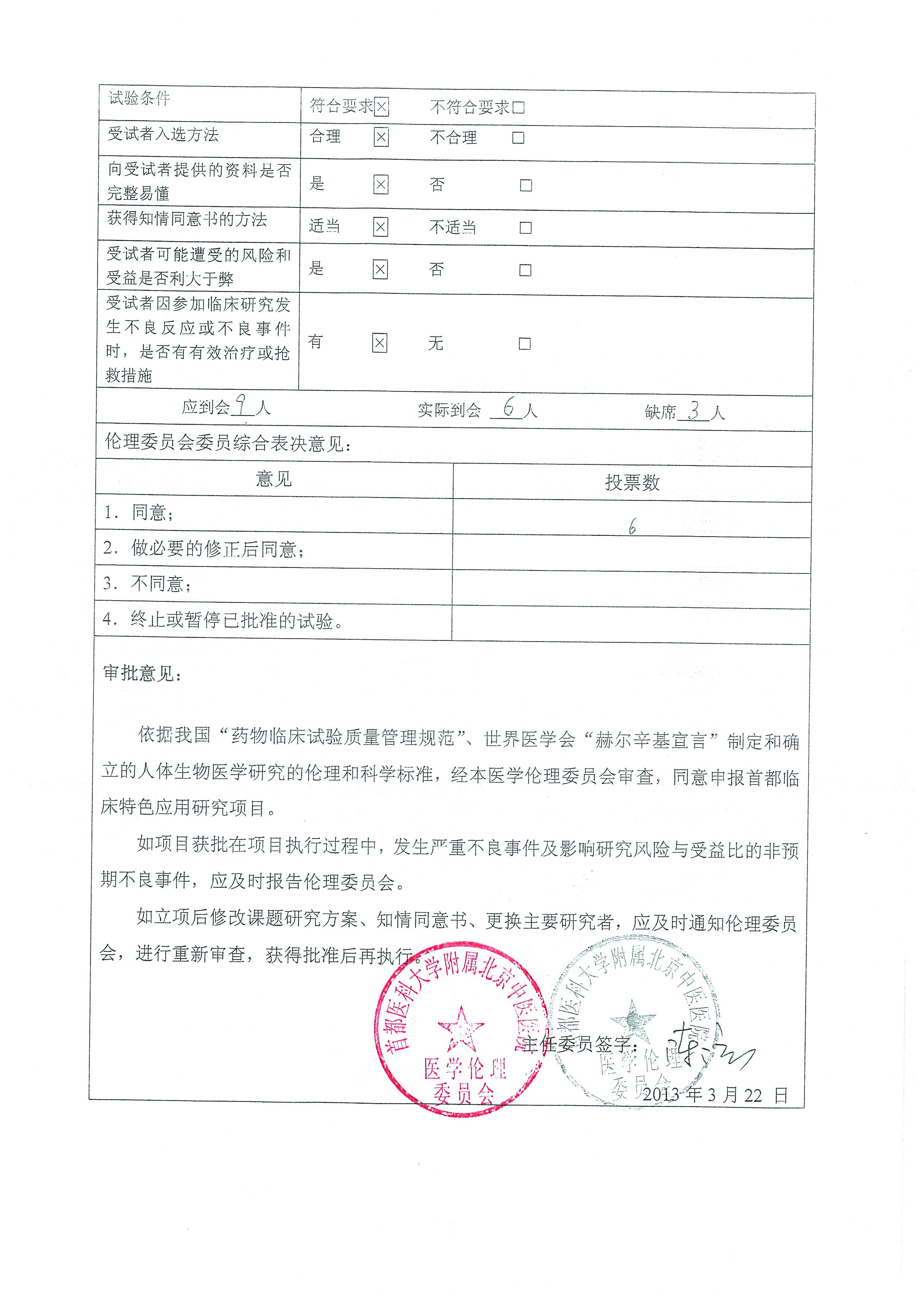

Supplement: Supplementary file 1 — Additional file 1: Ethical approval. (DOC 4 MB) [file 13063_2014_2306_MOESM1_ESM.doc]
